# miR-15b reduces amyloid-β accumulation in SH-SY5Y cell line through targetting NF-κB signaling and BACE1

**DOI:** 10.1042/BSR20180051

**Published:** 2018-11-14

**Authors:** Juan Li, Haitao Wang

**Affiliations:** 1 Department of Geriatrics, Zhejiang Integrated Chinese and Western Medicine Hospital, Hangzhou 310006, P.R. China; 2 Department of Neurology, Wuhan Union Hospital, Attending Physician, Wuhan 430022, P.R. China

**Keywords:** Aβ, Alzheimer’s disease, BACE1, miR-15b, NF-κB

## Abstract

Alzheimer’s disease (AD) is the multifactorial neurodegenerative disorder causing progressive memory loss and cognitive impairment. The aberrant accumulation of amyloid-β (Aβ) and neuroinflammation are two major events in AD. BACE1 is required for the cleavage of amyloid precursor protein (APP) to generate Aβ, which stimulates the nuclear transcription factor κB (NF-κB) signaling, leading to the secretion of inflammatory cytokines. And NF-κB can up-regulate the expression of BACE1. miRNAs are small non-coding RNAs that regulate gene transcription. miR-15b down-regulates BACE1 expression while it is unclear whether miR-15b can regulate Aβ in human neuronal cells, and if so, whether it is by targetting NF-κB. SH-SY5Y cell line was transfected with Swedish APP mutant (APPswe) as an *in vitro* AD model. Quantitative PCR (qPCR), WB, and ELISA were used to detected related gene expression intracellularly or in supernatant. Dual luciferase assay was used to validate miRNA and targets binding. miR-15b inhibits expression of BACE1and APP. Moreover, the reduced level of Aβ was observed in response to miR-15b mimics in SH-SH5Y/APPswe cells. miR-15b directly targetted the conserved Bace1 3′UTR to regulate its expression. In addition, the inhibition of APPswe-induced secretion of inflammatory cytokines and the suppression of NF-κB activation by miR-15b were validated. And miR-15b directly targetted the 3′UTRs of NF-κB1 and inhibitor of NF-κB (IκB) kinase α (IKK-α), encoding NF-κB1 and IKK-α, respectively. Our study suggests that miR-15b inhibits Aβ accumulation through targetting NF-κB signaling and BACE1 and serves as a potential molecular target for AD therapy.

## Introduction

Alzheimer’s disease (AD) is one of the most common neurodegenerative disorders associated with multifactors including the formation of amyloid-β (Aβ) plaques, τ hyperphosporylation, microglia activation, and neurone degeneration. Patients with AD often show progressive memory loss and cognitive impairment. Current therapies for AD are only symptomatic and none are able to delay or prevent disease development. Although AD is caused by many factors, the abnormal accumulation of Aβ is still the central event in the onset and progression of AD. Aβ is generated by the sequential cleavage of amyloid precursor protein (APP) by β-secretases, such as BACE1, and γ-secretase complex. Aβ_40_ and Aβ_42_ are two major species with Aβ_42_ being the major component of Aβ plaques in the brain of AD patients [1–4]. In the recent decade, people are focussing on the discovery of specific BACE1 and γ-secretase inhibitors to reduce the generation of Aβ and treat AD. However, both secretases are involved in maintaining various physiological processes and the inhibition of enzyme activity could bring tremendous side effects. So far, none of secretase inhibitors has been approved for clinical use and novel drug targets are desperately required for AD therapy.

miRNAs are small endogenous non-coding RNAs that recognize specific gene sequences located within the 3′UTRs thereby regulating gene expression. Mounting miRNAs have been implicated in the pathology of various diseases including cancers and neurodegenerative disorders [5–7]. A variety of miRNAs are expressed in the brain and involved in the regulation of biological functions as well as disease processes such as AD [8,9]. miR-98-5p is up-regulated in AD patients [10] and promotes BACE1 expression and Aβ level in neuroblastoma cell line [11]. In contrast, miR-124-3p is down-regulated in AD cell model and able to attenuate cell apoptosis and inhibit τ hyperphosphorylation [12]. Thus, the regulation of miRNAs might be an alternative approach to treat AD. miR-15b, a member of miR15/107 family, is dysregulated in various human cancers and the reduced expression of miR-15b is noticed in AD [13]. miR-15b can down-regulate BACE1 expression in rat dorsal root ganglion (DRG) neurones by targetting the 3′UTR of *Bace1* [14]. Therefore, we suspect that miR-15b could also target BACE1 in progress of AD.

In addition to Aβ pathology, the role of inflammation is now increasingly appreciated in AD development. Nuclear transcription factor κB (NF-κB) is a ubiquitously expressed transcription factor controlling the expression of numerous genes involved in inflammation. It is a complex of dimeric subunits that belong to the NF-κB/Rel family containing p50 (NF-κB1), p52 (NF-κB2), p65, c-Rel, and RelB. NF-κB is bound to inhibitor of NF-κB (IκB) and retained in the cytoplasm in an inactive state. A variety of extracellular stimuli, such as Aβ, induce the phosphorylation and subsequent degradation of IκB, which leads to the translocation of NF-κB into the nucleus, regulating the gene transcription of its target genes [15]. Aβ-induced activation of NF-κB contributes to the up-regulation of pro-inflammatory cytokines, such as tumor necrosis factor (TNF)-α and interleukin (IL)-6, and pro-inflammatory proteins, like cyclooxygenase-2 (COX-2) and inducible nitric oxide synthase (iNOS), leading to inflammatory response and cell death [16]. Interestingly, NF-κB activation can stimulate the expressions of BACE1 and APP [17]. Therefore, the present study is designed to elucidate the role of miR-15b in the modulation of NF-κB and BACE1 which may provide a novel therapeutic target for AD.

## Materials and methods

### Reagents and antibodies

All chemicals and reagents used here were purchased from Sigma (St. Louis, MO, U.S.A.) unless otherwise indicated. Anti-APP, anti-BACE1, anti-sAPPα, and anti-sAPPβ antibodies were from Abcam (Cambridge, U.K.). Anti-COX-2, anti-iNOS, anti-p-p65, anti-p-65, anti-p-IκBα, anti-IκBα, anti-IKK-α (IκB kinase α), anti-NF-κB1 p105, anti-NF-κB1 p50, and anti-β-actin antibodies were from Cell Signaling Technology (Danvers, MA, U.S.A.).

### Cell culture

SH-SY5Y and HEK293T were purchased from ATCC and maintained in Dulbecco’s modified Eagle’s medium with 10% (*v/v*) heat-inactivated FBS in a humidified incubator with 5% CO_2_/95% (*v/v*) air at 37°C. The Swedish APP mutant (APPswe) was transfected into SH-SY5Y cells which were selected with antibiotics (G418) and maintained in lab.

### Plasmids

The human *Bace1, NFκB1*, and *IKK-α* 3′UTR reporter luciferase constructs were made as per previous reports [14,18,19]. Briefly, the sequences of 3′UTR *Bace1, NFKB1*, and *IKK-α* were amplified from HEK293T cDNA and cloned into pMIR-basic vector (Promega, Madison, WI). All constructs were verified by sequencing.

### Transfection

Human mature has-miR-15b mimics, an inhibitor, and respective negative control (NC) were designed and synthesized by Dharmacon (Lafayeet, CO). They and the plasmids were transfected using X-tremeGENE HP DNA transfection reagent (Roche, CA, U.S.A.) following the manufacturer’s instructions. Cells were harvested for corresponding experiments at 24 or 48 h post-transfection.

### Quantitative PCR

Total RNA was extracted from SH-SY5Y with TRI Reagent (T9424) following the manufacturer’s instructions. Reverse transcription was then performed using random hexamer primer and MMLV Reverse Transcriptase (M5301; Promega). Quantitative PCR (qPCR) for the quantitation of gene transcripts was performed with 2× HotStart SYBR Green qPCR Master Mix (TransGen Biotech, Beijing, China) using a Stratagene Mx3000P (Agilent Technologies). The primer sequences are as below. miR-15b, sense: 5′-CTTCTGTCTATCACATAAGTGG-3′; antisense: 5′-GGTCCAAGTCAATTC CATG-3′; APP, sense: 5′-TGGCCCTGGAGAACTACATC-5′; antisense: 5′-AAT CACACGGAGGTGTGTCA-3′; BACE1, sense: 5′-TCTGTCGGAGGGAGCATG AT-3′; antisense: 5′-GCAAACGAAGGTTGGTGGT-3′; TNF-α, sense: 5′-GGA ACACGTCGTGGGATAATG-3′; antisense: 5′-GGCAGACTTTGGATGCTT CTT-3′; IL-1β, sense: 5′-TCCAGGATGAGGACATGAGCAC-3′; antisense: 5′-GAACGTCACACACCAGCAGGTTA-3′; IL-6, sense: TGTATGAACAACGATGATGCACTT; antisense: ACTCTGGCTTTGTCTTTCTTGTTATCT; COX-2, sense: 5′-GGGGTA CCTTCCAGCTGTCAAAATCTC-3′; antisense: 5′-GAAGATCTCGCCAGG TACTCACCTG-3′). iNOS, sense: 5′-CCCTTCCGAAGTTTCTGGCAGCAGC-3′; antisense: 5′-GGCTGTCAGAGCCTCGTGGCTTTGG-3′.

### Western blot analysis

Cells were lysed using radioimmunoprecipitation assay (RIPA) buffer (25 mmol/l Tris/HCl (pH 7.6), 150 mmol/l NaCl, 1% NP-40, 1% sodium deoxycholate) with 0.01% protease and phosphatase inhibitors (Roche). Protein levels were quantitated by a BCA Protein Assay Kit (Thermo Fisher Scientific). Protein lysates were separated by 10% SDS/PAGE and transferred on to nitrocellulose membranes (Millipore). Membranes were incubated with relevant antibodies at 4°C overnight followed by HRP–conjugated secondary antibody. The proteins were visualized by ECL reagent (Thermo Fisher Scientific). β-actin was used as a loading control.

### ELISA

The culture medium for 24 h from SH-SY5Y/APPswe or SH-SY5Y cells was collected and subjected to sandwish ELISA assay for the detection of Aβ_40_, Aβ_42_, TNF-α, IL-1β, IL-6, or prostaglandin E2 (PGE2) according to the manufacturer’s instructions (Life Technologies).

### Dual-Glo luciferase assay

HEK 293T cells were seeded into 12-well plate at 10^5^ cells/well. On the next day, cells were co-transfected with a NC, miR-15b mimics or inhibtor, *Renilla* luciferase plasmid, and pMIR plasmid harboring 3′UTR of *Bace1* (wild-type (WT) or mutant) or pMIR plasmid encoding *NFKB1* or *IKK-α* (WT or mutant). At 24 h post-transfection, luciferase activities were determined using a Dual-Glo Luciferase Assay System (Promega, Madison, WI) following manufacturer’s protocol. Firefly luciferase activity was normalized to *Renilla* luciferase activity.

### Statistical analysis

Each experiment was repeated at least three times. Data shown here are either representative or mean +/± S.E.M.. Data were analyzed by GraphPad Prism 6.0 (GraphPad Software Inc., San Diego, CA) and statistical analysis was performed using one-way or two-way ANOVA for multiple comparisons or unpaired *t* test for two-group comparisons. *P*<0.05 was considered significant. *, *P*<0.05; **, *P*<0.01; ***, *P*<0.001.

## Results

### miR-15b regulates the expression of APP and BACE1 in SH-SY5Y cells

**Figure 1:**
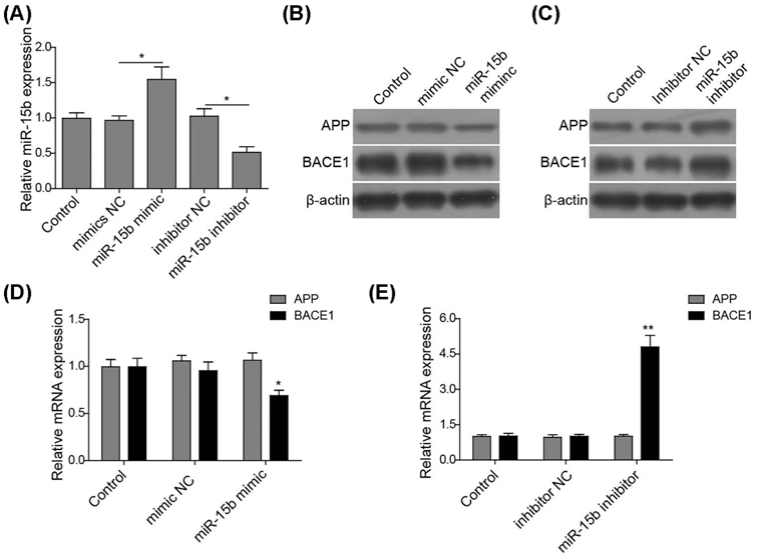
miR-15b regulates the expression of both APP and BACE1 in SH-SY5Y cells (**A**) The level of miR-15b in SH-SY5Y cells transfected with control vector, miR-15b mimic, mimic NC, miR-15b inhibitor, or inhibitor NC. Data are normalized to the control. (**B**) Representative image showing the protein expressions of APP and BACE1 in the cells transfected with control vector, miR-15b mimic, or mimic NC. β-actin was used as loading control. (**C**) Representative image showing the protein expressions of APP and BACE1 in the cells transfected with control vector, miR-15b inhibitor, or inhibitor NC. β-actin was used as loading control. (**D**) The mRNA level of APP and BACE1 in the cells transfected miR-15b mimic, or mimic NC. Data are normalized to the corresponding control. (**E**). The mRNA level of APP and BACE1 in the cells transfected with miR-15b inhibitor, or inhibitor NC. Data are normalized to the corresponding control. *P<0.05, **P<0.01 compared with respective NC group.

miR-15b has been revealed to down-regulate BACE1 expression in rat DRG neurones [14]. Here, experiments were designed to investigate the role of miR-15b on both BACE1 and APP expressions in a human neuronal cell line SH-SY5Y. Cells were transiently transfected with miR-15b mimic, miR-15b inhibitor, or their corresponding NC. qPCR data validated that the introduction of miR-15b mimic significantly enhanced the mRNA level of miR-15b while the presence of miR-15 inhibitor reduced miR-15b expression compared with the control (Figure 1A). The respective effects of mimic or inhibitor on the expressions of BACE1 and APP were investigated. miR-15b mimic suppressed the protein expressions of BACE1 and APP (Figure 1B). On the contrary, the inhibition of miR-15b promoted both BACE1 and APP levels (Figure 1C), whether miR-15b transcriptionally regulates BACE1 and APP mRNA expression was then determined. Interestingly, the expression of miR-15b mimic significantly reduced the mRNA level of BACE1 but not that of APP (Figure 1D). Consistently, the inhibition of miR-15b increased *BACE1* mRNA level while the expression of APP remained unchanged (Figure 1E). All these suggest that miR-15b can transcriptionally regulate BACE1 expression in human neuronal cells.

### miR-15b inhibits Aβ_40_ and Aβ_42_ accumulation in SH-SY5Y/APPswe cells

**Figure 2:**
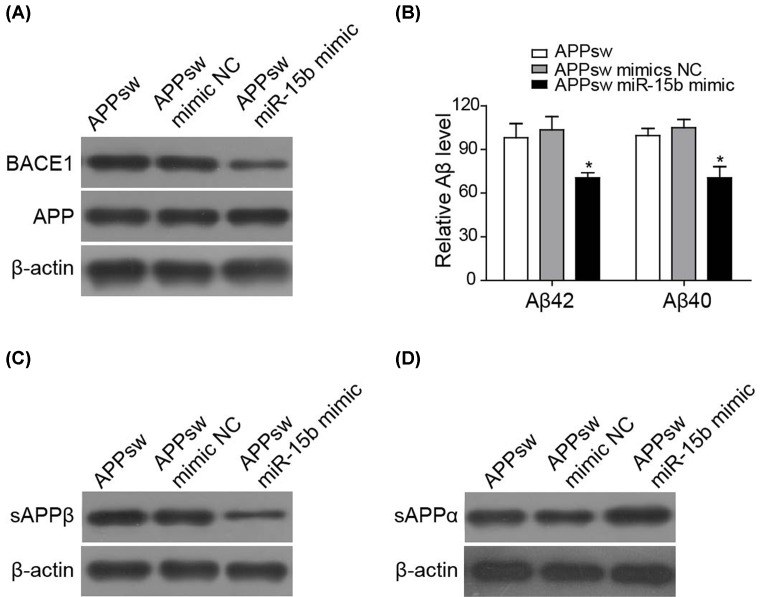
miR-15b inhibits Aβ_40_ and Aβ_42_ accumulation in SH-SY5Y/APPswe cells (**A**) Representative image demonstrating the protein level of BACE1 and APP in SH-SY5Y/APPswe cells transfected with control vector, mimic NC, or miR-15b mimic. (**B**) Aβ_40_ and Aβ_42_ levels in the culture medium from SH-SY5Y/APPswe cells transfected with control vector, mimic NC, or miR-15b mimic. Representative images showing the level of sAPPβ (**C**) or sAPPα (**D**) in the culture medium. β-actin was used as the loading control. *P<0.05.

The expression of BACE1 would directly influence the accumulation of Aβ. To test this, SH-SY5Y transiently transfected with APPswe mutant were used as an *in vitro* AD neuronal cell model. As expected, the expression of miR-15b mimic inhibited the expression of BACE1 in SH-SY5Y/APPswe cell, without an obvious effect on APP level (Figure 2A). This may be explained as the cells were transfected with overexpressed APPswe. Simultaneously, the level of either Aβ_40_ or Aβ_42_ in the culture medium was markedly reduced in the cells with miR-15b mimic expression (Figure 2B). APP is cleaved by α-secretase or BACE1 to produce soluble extracellular fragment of sAPPα or sAPPβ, respectively. The reduced level of sAPPβ accompanied with the increased sAPPα was observed in response to miR-15b mimic transfection which indicates the down-regulation of BACE1 by miR-15b (Figure 2C,D).

### miR-15b directly targets the conserved *Bace1* 3′UTR sequence to regulate its expression

**Figure 3:**
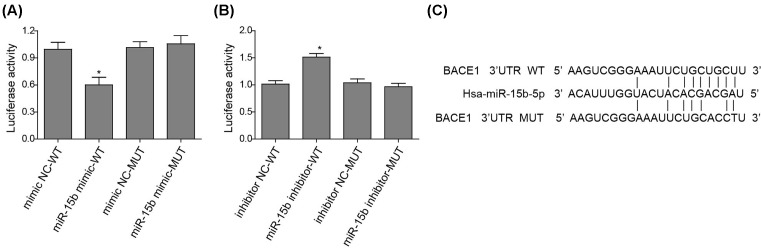
miR-15b directly targets the conserved *Bace1* 3′UTR sequence (**A**) The luciferase activity of wild-type (WT) or mutant (MUT) *Bace1* 3′UTR in the HEK 293T cells transfected with an NC or miR-15b mimic. Data are normalized to the WT mimic NC. (**B**) The luciferase activity of WT or MUT *Bace1* 3′UTR in the HEK 293T cells transfected with an NC or miR-15b inhibitor. Data are normalized to the WT inhibitor NC. (**C**) The schematic representation of miR-15b and its target sequence (WT or MUT) within the *Bace1* 3′UTR of mammals. *P<0.05 compared with respective NC group.

Since miRNAs inhibit transcription of certain mRNAs by binding to their 3′UTR sequences, we then determined whether miR-15b can target the 3′UTR of *Bace1* by luciferase reporter assay. The luciferase activity of *Bace1* 3′UTR was attenuated by the expression of miR-15b mimic, which was undetected when the mutant*Bace1* 3′UTR expressed (Figure 3A). On the contrary, the inhibition of miR-15b promoted *Bace1* 3′UTR luciferase activity which was not observed under mutant *Bace1* 3′UTR expression (Figure 3B). miR-15b targetting sequence of the *Bace1* 3′UTR is showed in Figure 3C. This result implied that miR-15b can directly target the conserved *Bace1* 3′UTR sequence to regulate its expression.

### miR-15b suppresses the secretion of inflammatory cytokines through inhibition of NF-κB signaling

**Figure 4:**
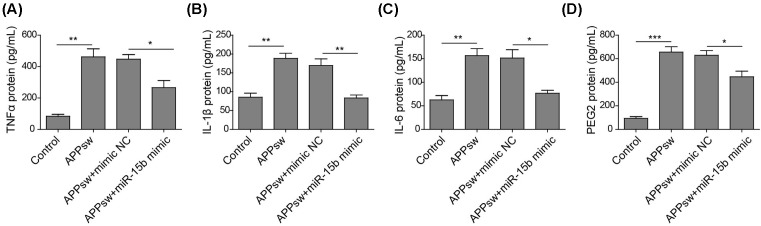
miR-15b inhibits APPswe-induced secretion of inflammatory cytokines The levels of TNF-α (**A**), IL-1β (**B**), IL-6 (**C**), and PGE2 (**D**) in the culture medium of SH-SY5Y cells either transfected with control vector or APPswe plasmid alone or co-transfected with APPswe and an NC or miR-15b mimic. *P<0.05, **P<0.01, ***P<0.001.

**Figure 5:**
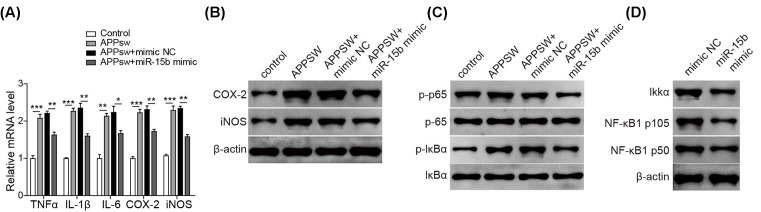
miR-15b inhibits APPswe-induced expression of inflammatory products via suppressing NF-κB signaling pathway (**A**) The mRNA levels of TNF-α, IL-1β, IL-6, COX-2, and iNOS in SH-SY5Y cells either transfected with control vector or APPswe plasmid alone or co-transfected with APPswe and mimic NC or miR-15b mimic. (**B**) The protein levels of COX-2 and iNOS in the cells either transfected with control vector or APPswe plasmid alone or co-transfected with APPswe and an NC or miR-15b mimic. β-actin was used as the loading control. (**C**) The protein levels of p-p65, p-65, p-IκBα, and IκBα in the cells transfected with control vector or APPswe alone or co-transfected with APPswe and mimic NC or miR-15b mimic. (**D**) The protein levels of IKK-α, NF-κB1, p105, and NF-κB1 p50 in the cells transfected with mimic NC or miR-15b mimic. β-actin was used as the loading control. *P<0.05, **P<0.01, ***P<0.001.

Aβ stimulates the secretion of inflammatory cytokines and induces inflammatory responses. Here, the introduction of APPswe into SH-SY5Y cells induced the secretion of pro-inflammatory cytokines including TNF-α, IL-1β, IL-6, and PGE2 (Figure 4). Interestingly, the APPswe-induced secretion of these pro-inflammatory cytokines was reduced by the overexpression of miR-15b. Moreover, the increased mRNA levels of TNF-α, IL-1β, IL-6, COX-2, and iNOS were all observed in response to the transfection with APPswe, which was significantly suppressed by miR-15b mimic (Figure 5A). In addition, the expression of APPswe increased the expressions of COX-2 and iNOS at protein levels and this effect was reduced when miR-15b mimic co-expressed (Figure 5B). All these indicate that miR-15b can inhibit Aβ-induced secretion and expression of pro-inflammatory cytokines.

It is known that Aβ can stimulate inflammatory cytokines secretion through the activation of NF-κB signaling. Here, the transfection with APPswe resulted into the enhanced expression of phosphor-p65 (p-p65) and phosphor-IκBα (p-IκBα) without the change in total p65 and IκBα levels, indicating the activation of NF-κB (Figure 5C). This effect was inhibited by the co-transfection with miR-15b mimic. Furthermore, treatment with miR-15b mimic induced the down-regulation of IKK-α, NF-κB p105, and NF-κB p50 subunit without the influence of β-actin expression (Figure 5D).

**Figure 6:**
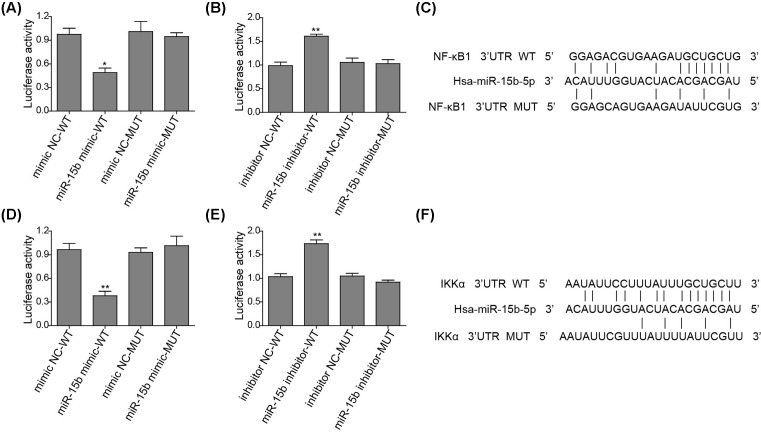
miR-15b directly targets the 3′UTR sequences of *IKK-a* and *NFKB1* (**A**,**B**) WT or MUT *NFKB1* reporter plasmid were co-transfected with a respective NC or miR-15b mimic (A) or inhibitor (B) into HEK293T cells. The luciferase activity was measured. Data are normalized to the WT mimic NC. (**C**) The schematic representation of has-miR-15b-5p and its target sequence (WT and MUT) within the *NFKB1* 3′UTR. (**D**,**E**) WT or MUT *IKK-a* reporter plasmid were co-transfected with a respective NC or miR-15b mimic (D) or inhibitor (E). The luciferase activity was measured. Data are normalized to the control with WT *IKK-a*. (b) The schematic representation of has-miR-15b-5p and its target sequence (WT and MUT) within the *IKK-a* 3′UTR. *P<0.05, **P<0.01.

One recent study identified *NFKB1* and *IKK-*α as putative targets of miR-15b [19]. Here, the transfection with miR-15b mimics significantly reduced the reporter activity of NF-κB1 and IKK-α (Figure 6A,D). As reported, putative binding sites for miR-15b were identified in the 3′UTRs of *NFKB1* and *IKK-α* (Figure 6C,F). miR-15b failed to regulate either NF-κB1 or IKK-α transcription when the mutation within the corresponding 3′UTRs sequence applied (Figure 6A,D). By contrast, the introduction of miR-15b inhibitor promoted the reporter activity of NF-κB1 and IKK-α (Figure 6B,E). This was not observed in the cells introduced by the corresponding mutant. These data suggest that miR-15b suppresses inflammatory cytokine secretion through targetting NF-κB signaling.

## Discussion

Although AD is a multifactorial disorder, the abnormal accumulation of Aβ is still the central event. β-secretase, especially BACE1 which is highly expressed in neurones, is responsible for the generation of Aβ by the cleavage of APP and both the expression and the activity of BACE1 are enhanced in the brains of AD patients [20,21]. Therefore BACE1 has been a drug target for AD therapy. This is supported by the studies showing either deletion or inhibition of BACE1 precludes Aβ pathology and cognitive impairment in AD mouse model [22,23]. The interest for miRNA replacement therapy is rapidly growing. Compared with small molecular inhibitor, miRNA is endogenously expressed in cells, thus, off-target effects are less likely to occur as the mimics behave like their natural counterparts by fine-tuning the expression of targets. Another strong rational to use miRNAs in replacement therapy is based on the fact that a single miRNA can regulate multiple genes simultaneously, providing a synthetic regulation for treatment [24]. Here, we presented that miR-15b can regulate BACE1 expression and Aβ accumulation in human neuronal cells, providing an alternative strategy for the treatment of AD.

miRNAs are highly expressed in the brain which fine-tune the expression of their target genes and regulate multiple biological events. Previous studies have implicated that several miRNAs are involved in the development of AD through the regulation of BACE1 expression or τ hyperphosphorylation [11,12,25]. The role of miR-15/107 family in the regulation of cancers, cardiovascular disorders, and neurodegenerative diseases is increasingly appreciated [26]. As a member, miR-15b is highly expressed in various tumors and critical in cancers [27,28]. It targets the genes involved in cell proliferation and apoptosis [29,30]. miR-15b is also implicated in AD and has been reported to target *Bace1* 3′UTR and regulate BACE1 expression in rat DRG neurone [13,14]. However, whether miR-15b shows the similar effect in human neuronal cells and regulates Aβ accumulation remains to be clarified. Our study reveals a consistent inhibitory effect of miR-15b on the modulation of BACE1 expression in human neuroblastoma cell line and further demonstrates the role of miR-15b in the reduction in APP and Aβ levels in a human AD cell model. These further indicate that miR-15b is involved in AD and this miRNA could be a novel therapeutic target for AD treatment. Since first miRNA therapy entering phase 1 clinical trial, miRNAs have been extensively studied as a promising therapeutic strategy including neurodegenerative diseases. However, RNAi-based therapeutics often face *in vivo* instability due to enzyme degradation and non-regenerating neuronal cells also represent difficulties for delivery of engineered miRNAs in CNS. Viral and non-viral carrier systems such as Lipid-based carriers or Gold Nanoparticles have been developed for miRNA delivery and some advancement has been achieved. Our further investigation will focus on finding proper carriers for miR-15b delivery in AD [31].

NF-κB regulates the expression of its target genes involved in inflammation. NF-κB signaling is activated in AD and mediates inflammatory responses induced by Aβ. Thus, NF-κB is considered as a potential target for the treatment of AD [32]. NF-κB1, including p105 and p50, is one of the subunits of NF-κB. IKK-α phosphorylates IκB, leading to the degradation and subsequent activation of gene transcription by NF-κB. Studies have indicated the role of miRNAs in the regulation of NF-κB signaling [19,33]. Both the recent and the current study identify *NFKB1* and *IKK-α*, encoding NF-κB1 and IKK-α, as targets of miR-15b which negatively regulate the expression of NF-κB1 and IKK-α [19] thereby suppressing NF-κB activation. As demonstrated in the present study, this could be a novel mechanism for miR-15b to regulate inflammatory response in response to Aβ. Moreover, BACE1 and APP are also transcription targets of NF-κB. miR-15b down-regulates BACE1 and APP expression possibly through targetting NF-κB. All these indicate the protective role of miR-15b in AD development. In detail, miR-15b exerted protective effect by two possible mechanism: inhibiting the inflammation reaction in AD and attenuating the expression of BACE1 via direct and indirect way.

Further mechanism remains unclear in this regulatory axis. It is known that miR-15b can be transcriptionally up-regulated by NF-κB component c-Rel [18]. Thus, the activation of NF-κB in AD may enhance miR-15b expression, which is demonstrated in the current study can in turn negatively regulate NF-κB signaling pathway, BACE1 expression, and Aβ accumulation. This may serve as a negative feedback in AD pathology, which could be an essential event in preventing disease progress in the early stage. The mechanistic insight of this feedback circuit requires further investigation.

Though it is reported that miR-15b has pro-tumor properties, we noticed that this tumorigenesis effect is related to abnormal high expression of miR-15b in cancer, while in AD progress, miR-15/107 family were down-regulated below physiological level [24]. This may indicate that if restoration of miR-15b level is controlled within normal level, the intervention of miR-15b for AD treatment may not cause unfavorable effect. Further researches should be performed on to understand the threshold copies of miR-15b that should be restored or repressed in each disease state. Additionally, local delivery of miR-15b into the hippocampus and cortex via novel carrier mentioned above may also shed a light on preventing detrimental effect of miRNA treatment.

## Abbreviations

AD: Alzheimer’s disease
APP: amyloid precursor protein
APPswe: Swedish APP mutant
Aβ: amyloid-β
COX-2: cyclooxygenase-2
DRG: dorsal root ganglion
IKK-α: inhibitor of NF-κB kinase α
IL: interleukin
iNOS: inducible nitric oxide synthase
IκB: inhibitor of NF-κB
NF-κB: nuclear transcription factor κB
NC: negative control
PGE2: prostaglandin E2
qPCR: quantitative PCR
TNF: tumor necrosis factor
