# Expression of Concern: miR-15b Reduces Amyloid-β Accumulation in SH-SY5Y Cell Line through Targeting NF-κB signaling and BACE1

**DOI:** 10.1042/BSR-20180051_EOC

**Published:** 2021-04-09

**Keywords:** Aβ, Alzheimer's disease, BACE1, miR-15b, NF-κB

The Editorial Office has been made aware of potential issues surrounding the scientific validity of this paper, hence has issued an expression of concern to notify readers whilst the Editorial Office investigates.

